# Mitogen-Activated Protein Kinase-Activated Protein Kinase 2 Deficiency Reduces Insulin Sensitivity in High-Fat Diet-Fed Mice

**DOI:** 10.1371/journal.pone.0106300

**Published:** 2014-09-18

**Authors:** Jan Freark de Boer, Arne Dikkers, Angelika Jurdzinski, Johann von Felden, Matthias Gaestel, Udo Bavendiek, Uwe J. F. Tietge

**Affiliations:** 1 Department of Pediatrics, Center for Liver, Digestive and Metabolic Diseases, University of Groningen, University Medical Center Groningen, Groningen, The Netherlands; 2 Clinic of Cardiology and Angiology, Hannover Medical School, Hannover, Germany; 3 Institute of Biochemistry, Hannover Medical School, Hannover, Germany; Tohoku University, Japan

## Abstract

Adipose tissue inflammation is considered an important contributor to insulin resistance. Mitogen-activated protein kinase-activated protein kinase 2 (MK2) is a major downstream target of p38 MAPK and enhances inflammatory processes. In line with the role of MK2 as contributor to inflammation, *MK2^−/−^* mice are protected against inflammation in different disease models. Therefore, MK2 is considered an attractive therapeutic target for the treatment of chronic inflammatory diseases. This study tested the impact of MK2-deficiency on high-fat diet (HFD)-induced adipose tissue inflammation and insulin resistance. After feeding *MK2^−/−^* and WT control mice a HFD (60% energy from fat) for 24 weeks, body weight was not different between groups. Also, liver weight and the amount of abdominal fat remained unchanged. However, in *MK2^−/−^* mice plasma cholesterol levels were significantly increased. Surprisingly, macrophage infiltration in adipose tissue was not altered. However, adipose tissue macrophages were more skewed to the inflammatory M1 phenotype in *MK2^−/−^* mice. This differerence in macrophage polarization did however not translate in significantly altered expression levels of *Mcp-1*, *Tnfα* and *Il6*. Glucose and insulin tolerance tests demonstrated that *MK2^−/−^* mice had a significantly reduced glucose tolerance and increased insulin resistance. Noteworthy, the expression of the insulin-responsive glucose transporter type 4 (GLUT4) in adipose tissue of *MK2^−/−^* mice was reduced by 55% (p<0.05) and 33% (p<0.05) on the mRNA and protein level, respectively, compared to WT mice. *In conclusion*, HFD-fed *MK2^−/−^* display decreased glucose tolerance and increased insulin resistance compared to WT controls. Decreased adipose tissue expression of GLUT4 might contribute to this phenotype. The data obtained in this study indicate that clinical use of MK2 inhibitors has to be evaluated with caution, taking potential metabolic adverse effects into account.

## Introduction

As a result of the present obesity epidemic, prevalence of insulin resistance and type 2 diabetes mellitus is increasing rapidly in developed countries [1]. Inflammation most likely contributes to the development of insulin resistance. Altough inflammation might not impact on insulin sensitivity in the onset phase of obesity [2], inflammation within adipose tissue has been shown to have deleterious effects on systemic insulin sensitivity in models of chronic obesity [3]–[5]. Although many diffent types of immune cells are present in inflamed adipose tissue, macrophages are the major cell type associated with adipose tissue inflammation [4]. Interestingly, not only the amount of macrophages present in the adipose tissue increases with obesity, their phenotype also shifts. While anti-inflammatory M2 macrophages predominate in lean adipose tissue, the balance shifts towards more inflammatory M1 macrophages with increasing obesity [6]. Moreover, M1 macrophages are reported to negatively impact on insulin sensitivity compared to macrophages of the M2 phenotype [7]. Amelioration of adipose tissue inflammation might conceivably improve insulin sensitivity and thereby lead to a reduction of morbidity and mortality associated with type 2 diabetes.

A potential candidate pathway suitable for therapeutic intervention is the p38 mitogen-activated protein kinase (p38 MAPK, p38) pathway. Inhibition of p38 itself has potent anti-inflammatory effects. However, the wide range of biological effects of this signaling mediator hampers the clinical use of p38 inhibitors. Since p38 has numerous down-stream targets, inhibition of one of these targets might reduce adipose tissue inflammation without inducing substantial adverse effects. Mitogen-activated protein kinase-activated protein kinase 2 (Mapkapk2 or MK2) is a direct target of p38, enhances inflammatory processes and is essential for sustained activation of NF-κB, a central transcription factor in inflammation that has been shown to be involved in the development of insulin resistance [8], [9]. Furthermore, MK2 increases the mRNA stability of key proinflammatory cytokines, including TNFα, by phosphorylating tristetraprolin (TTP) which normally binds to the 3′-UTR of certain mRNA molecules and directs their deadenylation. However, upon phosphorylation by MK2, TTP is unable to recruit the deadenylation machinery, resulting in decreased mRNA degradation [10], and is replaced in RNA binding by the mRNA-stabilizing and –translation-stimulating factor HuR [11].

MK2 deficiency has been shown to result in a potent reduction of inflammation in several disease models and, so far, no severe side effects have been reported. It has for instance been shown that *MK2^−/−^* mice are resistant to endotoxic shock because of reduced production of TNFα after injection of lipopolysaccharide/D-galactosamine [12]. Furthermore, it has been demonstrated that *MK2^−/−^* mice have a reduced susceptibility for the development of collagen-induced rheumatoid arthritis (RA) [12] and that MK2^−/−^ mice on a *Ldlr^−/−^* background are protected against the development of atherosclerosis despite a pro-atherogenic lipoprotein profile [13]. The atheroprotective effect of MK2-deficiency observed in this study could be explained in part by a reduced expression of adhesion molecules and monocyte chemoattractant protein-1 (Mcp-1), factors that also play key roles in adipose tissue inflammation.

Therefore, the present study explored the effects of MK2-deficiency on the development of adipose tissue inflammation and insulin resistance in high-fat diet (HFD-) fed mice. In contrast to our hypothesis, no effect was observed in the amount of macrophages that had infiltrated the adipose tissue. The balance between M1 and M2 macrophages appeared, however, to be more skewed towards the M1 phenotype in MK2^−/−^ mice. HFD-fed *MK2^−/−^* mice were more insulin resistant compared to wild-type (WT) controls. Decreased expression of the glucose transporter GLUT4 in adipose tissue might contribute to the glucose intolerant, insulin resistant, phenotype observed in HFD-fed *MK2^−/−^* mice.

## Materials and Methods

### Animals

Male *MK2^−/−^* mice [12] were obtained from the breeding colony of Hannover Medical School and were backcrossed to the C57BL/6J genetic background for >10 generations using mice from Charles River (Sulzfeld, Germany). For the reported experiments littermate controls were used. All mice were exposed to a 12 hour light-dark cycle, were housed under climate-controlled conditions and had free access to food and water. Mice were fed a hypercaloric high-fat diet (HFD) containing 60% (energy) fat (AB-diets, Woerden, The Netherlands) for 24 weeks. After this period, experiments were carried out as indicated below. All animal experiments were performed according to the national law on animal welfare, and experimental procedures were approved by the responsible local ethics committee of the University of Groningen (Permit Number: 5997).

### Plasma lipid and lipoprotein analysis

Blood samples were obtained at the end of the study by cardiac puncture after 4 h of fasting using heparinized syringes, and were immediately placed on ice. Blood was centrifuged at 8000 rpm for 10 min at 4°C and plasma was stored at −80°C until further analysis. Plasma triglycerides, free fatty acids, glycerol, and cholesterol were determined using commercially available kits (Roche Diagnostics, Mannheim, Germany and Diagnostic Systems, Holzheim, Germany). Plasma insulin levels were measured with an ultrasensitive mouse insulin ELISA kit (Alpco, Salem, NH, USA). Pooled plasma samples were subjected to fast protein liquid chromatography (FPLC) gel filtration using a superose 6 column (GE Healthcare, Uppsala, Sweden) as described [14]. Individual fractions were assayed for cholesterol and triglyceride concentrations as detailed above.

### Analysis of gene expression

Gene expression was analyzed by real-time qPCR. Briefly, RNA was extracted from tissue samples with Tri-reagent (Sigma, St. Louis, MO, USA) and quantified with a NanoDrop ND-100 UV-Vis spectrophotometer (NanoDrop Technologies, Wilmington, DE, USA). One µg of RNA was reverse transcribed using M-MLV reverse transcriptase (Sigma) according to the manufacturer’s instructions. Real-time qPCR analysis was performed on a 7900 HT Fast Real-Time PCR system (Applied Biosystems, Darmstadt, Germany) using multi-exon spanning primer/probe sets synthesized by Eurogentec (Seraing, Belgium). Gene expression levels were normalized to cyclophilin and further normalized to the mean expression level of the control group. Gene expression levels of the macrophage polarization markers *Mgl-1* and *Mgl-2* were normalized to *Cd68* to correct for the amount of macrophages present in the adipose tissue.

### Glucose and insulin tolerance tests

Mice were fasted for 4 hours in the morning before the start of the glucose or insulin tolerance test. For the glucose tolerance test, mice were intraperitoneally (i.p.) injected with 1.25 g/kg glucose. Blood glucose levels were assessed by tail bleeding before injection and at 15, 30, 60 and 120 minutes after injection using a Onetouch Ultra glucose meter (LifeScan Benelux, Beerse, Belgium). The procedure for the insulin tolerance test was the same as described for the glucose tolerance test except that mice were injected with 0.6 U/kg insulin (i.p.) instead of glucose and blood glucose levels were measured at 15, 30, 45, 60 and 90 minutes after injection.

### Western blot

Adipose tissue samples were homogenized and sodium dodecyl sulfate (SDS) was added to a final concentration of 2%. Next, 25 µg protein of each sample was loaded onto a polyacrylamide gel for electrophoresis. Subsequently, semi-dry blotting was applied to transfer the proteins to a 0.45 µm nitrocellulose membrane (Schleicher & Schuell). Blocking of the membrane was performed overnight using 5% BSA in PBS after which the blot was incubated with an anti-GLUT4 antibody (Ab654, Abcam, Cambridge, UK) at a 1∶1500 dilution in PBS supplemented with 2% BSA and 0.1% Tween-20 for 2 h. A horseradish peroxidase-conjugated secondary goat anti-rabbit antibody (DAKO, Glostrup, Denmark) was then added at a 1∶2000 dilution for 45 min. Bands were visualized using chemiluminescence (GE Healthcare, Chalfont St Giles, UK). Band intensity was quantified using the freely available ImageJ software (http://rsbweb.nih.gov/ij/).

### Statistics

Statistical analyses were performed using the Statistical Package for Social Sciences (SPSS, SPSS Inc., Chicago, IL). Data are presented as means ± SEM. Differences between groups were compared using the Mann-Whitney U-test. For comparison between more than two groups, significance of differences was assessed using the Kruskal-Wallis test and Conover *post-hoc* analysis. P values <0.05 were considered statistically significant.

## Results

### MK2-deficiency does not affect body weight, but causes dyslipidemia in HFD-fed mice

After feeding a HFD for 24 weeks, *MK2^−/−^* mice and WT controls were sacrificed and blood and tissues were collected for analysis. Body weight of the mice did not differ between the groups and also liver weight was not different (Table 1). Furthermore, there was no effect of MK2 deficiency on the abdominal fat content of the mice (Table 1). However, plasma cholesterol levels were about 25% higher in *MK2^−/−^* mice compared to WT controls (Table 1). Plasma triglyceride levels tended to be increased as well in *MK2^−/−^* mice albeit this difference did not reach statistical significance (Table 1). Plasma free fatty acid levels were slightly decreased in *MK2^−/−^* mice (Table 1), whereas plasma glycerol levels remained unchanged (0.18±0.03 vs. 0.16±0.02 mmol/l). To identify which lipoproteins were responsible for the elevation of plasma lipids, lipoprotein subclasses were fractionated using Fast Protein Liquid Chromatography (FPLC). The cholesterol profile revealed that *MK2^−/−^* mice had a somewhat higher HDL peak. Furthermore, a shoulder was observed in the profile of *MK2^−/−^* mice, likely representing LDL particles (Fig. S1A). The triglyceride profile confirmed an increased amount of LDL particles and showed an increase in VLDL in the *MK2^−/−^* mice (Fig. S1B).

**Table 1:** Basal characteristics of HFD-fed WT and MK2^−/−^ mice.

	WT	MK2^−/−^
Body weight (g)	46.1±1.2	45.4±1.0
Liver weight (g)	2.8±0.3	2.4±0.2
Abdominal fat content (g)	4.9±0.2	4.7±0.1
Total plasma cholesterol (mM)	3.65±0.20	4.52±0.18**
Plasma triglycerides (mM)	0.46±0.03	0.68±0.08
Plasma free fatty acids (mM)	0.42±0.03	0.35±0.01*

*p<0.05,

**p<0.01 vs. WT controls.

Data are presented as means ± SEM (n = 8 animals per group).

### Impact of MK2-deficiency on adipose tissue inflammation

**Figure 1:**
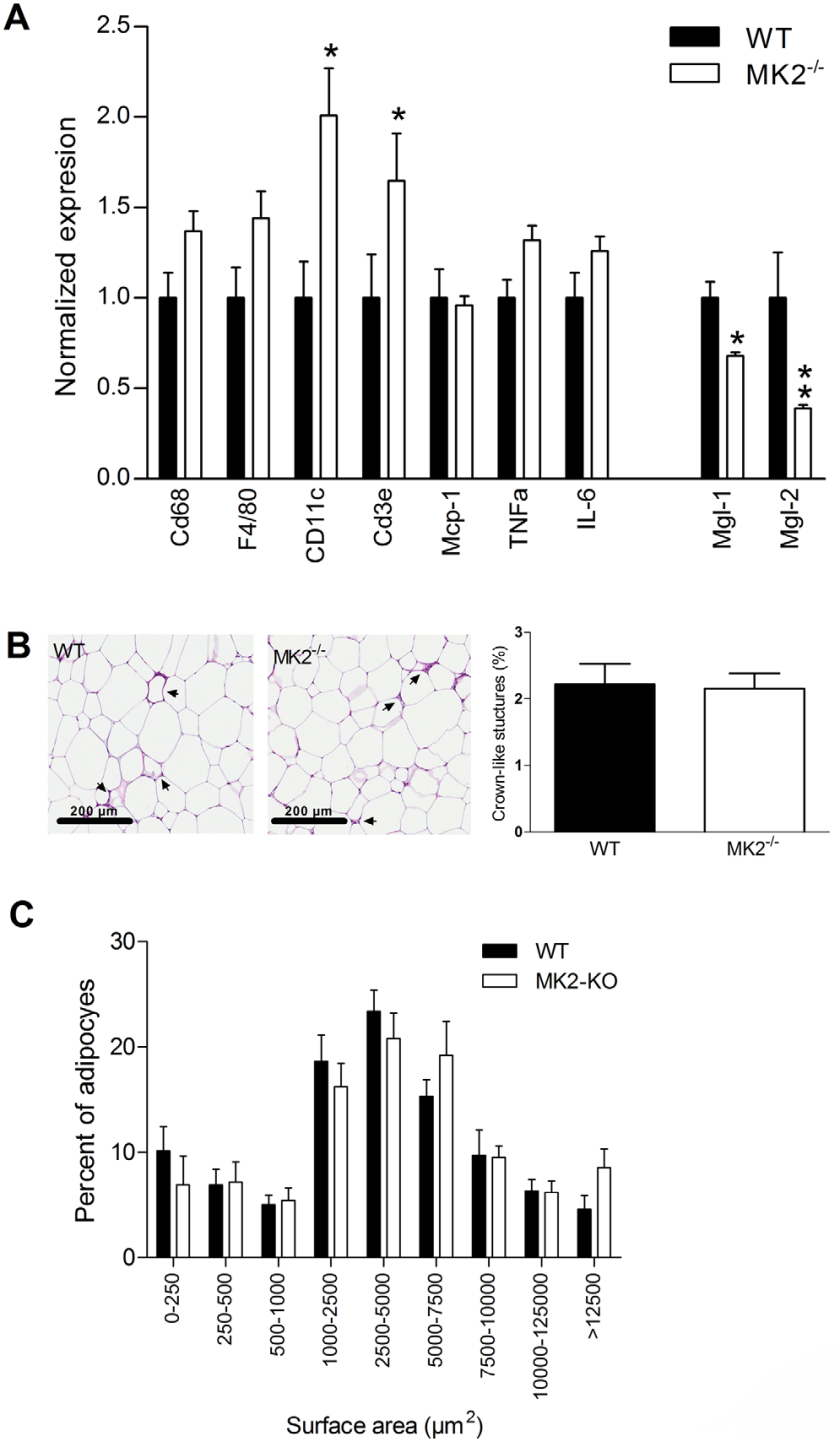
Impact of MK2-deficiency on adipose tissue inflammation. Abdominal fat was excised from mice that were fed a high-fat diet (HFD) for 24 weeks (n = 8 mice per group). Real-time qPCR was performed to measure mRNA expression of inflammation-related genes in adipose tissue (A). Formalin-fixed paraffin-embedded tissue sections were stained with hematoxyline and eosine to assess infiltration of immune cells in adipose tissue and sections from 5 randomly selected animals of each group were used to quantify the amount of crown-like structures. At least 300 adipocytes were counted per animal (B). Adipocyte size distribution was assessed by measuring the surface of ∼100 adipocytes per animal using freely available ImageJ software (imagej.nih.gov) and sections from 5 randomly selected animals of each group were used for quantification of adipocyte size (C).

Adipose tissue samples from 24 weeks HFD-fed mice were analyzed to assess the degree of inflammation. Compared to chow-fed animals, expression levels of macrophage markes (*Cd68* and *F4/80*) in adipose tissue were increased in both genotype (table S1), however there was no significant increase in the expression of macrophage markers between genotypes (Fig. 1A), indicating that the amount of adipose tissue macrophages was similar in both groups. Interestingly, the subpopulation of Cd11c-positive macrophages, which has been linked to insulin resistance, appeared to be increased in the adipose tissue of HFD-fed *MK2^−/−^* mice (Fig. 1A). Higher expression of *Cd3e* in the *MK2^−/−^* mice (Fig. 1A) suggests an increased presence of T cells in the adipose tissue of those mice. The polarization of adipose tissue macrophages appears to be altered in *MK2^−/−^* mice. HFD-feeding induced a shift in macrophage polarization towards the M1 phenotype as suggested by the decreased expression of the macrophage-specific M2-markers *Mgl-1* and *Mgl-2* (table S1). Specifically in the HFD-fed MK2^−/−^ mice, the expression of those markers was decreased compared to HFD-fed WT mice, suggestive of an altered macrophages polarization state in the adipose tissue of those mice. An increased M1/M2 macrophage balance is reported to result in more inflammation and to negatively impact insulin sensitivity [7]. The apparent increase in the M1/M2 balance did however not result in significantly altered expression levels of pro- as well as anti-inflammatory cytokines (Fig. 1A and table S1). Adipose tissue histology showed identical amounts of characteristic crown-like stuctures in the adipose tissue of the respective mouse models (Fig. 1B). In addition, no major differences in size distribution of adipocytes was observed (Fig. 1C). Analysis of liver histology did not reveal any difference in the degree of steatosis (Fig. S2). In addition, hepatic gene expression analysis did not show major differences in hepatic inflammation, although the amount of T cells might be somewhat higher in MK2^−/−^ mice compared to WT as indicated by the increased expression of the T cell marker *Cd3e*.

### MK2 deficiency results in impaired glucose tolerance and insulin resistance

**Figure 2:**
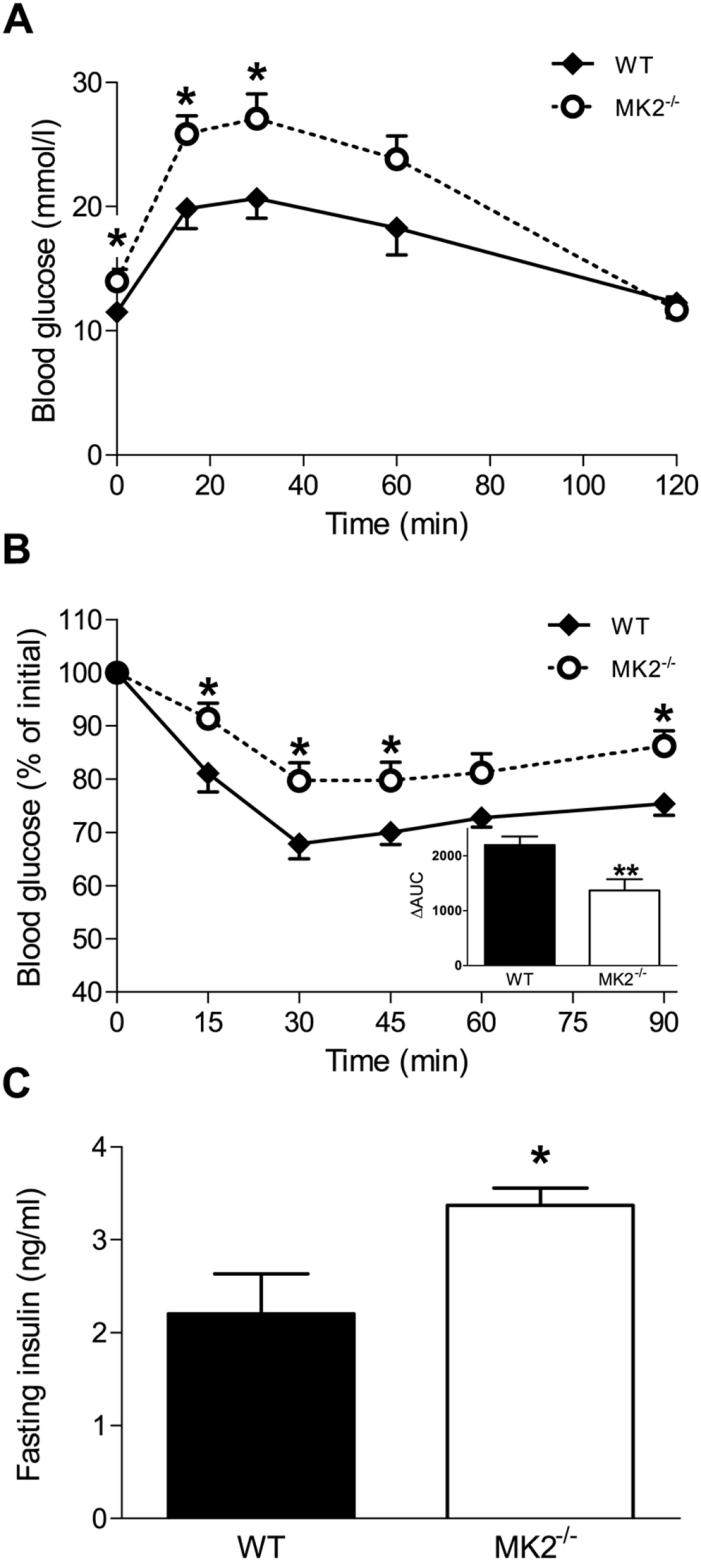
High-fat diet-fed *MK2* ^−/−^ mice are insulin resistant. High-fat diet (HFD) fed wild-type (WT) and *MK2^−/−^* mice were injected intraperitoneally (i.p.) with glucose (1.25 g/kg) and blood glucose levels were measured at the indicated time points (A). Several days later, mice were injected i.p. with insulin (0.6 U/kg) and blood glucose levels were determined at the indicated time points. Blood glucose levels during the insulin tolerance test (ITT) are depicted as percentage of initial blood glucose levels (B). ΔArea under the curve was calculated by subtracting the area under the curve of an individual mouse by the area under a hypothetical curve that remained at 100% throughout the duration of the test (B, insert). Fasting insulin levels were determined using an ultra-sensitive mouse insulin ELISA (C). *p<0.05, **p<0.01 compared to WT mice (n = 8 mice per group).

Chow-fed MK2^−/−^ mice displayed a moderately decreased glucose tolerance compared to WT controls but with identical fasting glucose levels (Fig. S3A). In the HFD-fed mice, fasting blood glucose levels were higher in MK2-deficient mice compared to WT (14.0±1.0 vs. 11.5±0.4 mmol/l, p<0.05, Fig. 2A). *MK2^−/−^* mice and WT controls were then injected i.p. with a glucose bolus (1.25 g/kg) to assess glucose tolerance. As indicated by the higher glucose levels during the glucose tolerance test, HFD-fed *MK2^−/−^* mice have an impaired glucose tolerance compared to WT controls (Fig. 2A), with significant differences at time points 15 and 30 minutes (both p<0.05). The difference in glucose tolerance remained significant when corrected for baseline glucose levels (Fig. S3B). Several days later, the mice were subjected to an insulin tolerance test to explore whether the impaired glucose tolerance in *MK2^−/−^* mice could be ascribed to insulin resistance. Higher blood glucose levels during the insulin tolerance test indicated that *MK2^−/−^* mice were indeed insulin resistant (Fig. 2B). Differences reached significance at time points 15, 30, 45 and 90 minutes (all p<0.05) and also the area under the curve (ΔAUC) was 38% reduced (p<0.01, Fig. 2B insert). Increased fasting plasma insulin levels in *MK2^−/−^* mice (+55%, p<0.05, Fig. 2C) further indicated that those mice were more insulin resistant compared to WT controls. Taken together, these data show that HFD-fed *MK2^−/−^* mice have a glucose intolerant, insulin resistant phenotype.

### GLUT4 expression is reduced in adipose tissue of HFD-fed MK2^−/−^ mice

**Figure 3:**
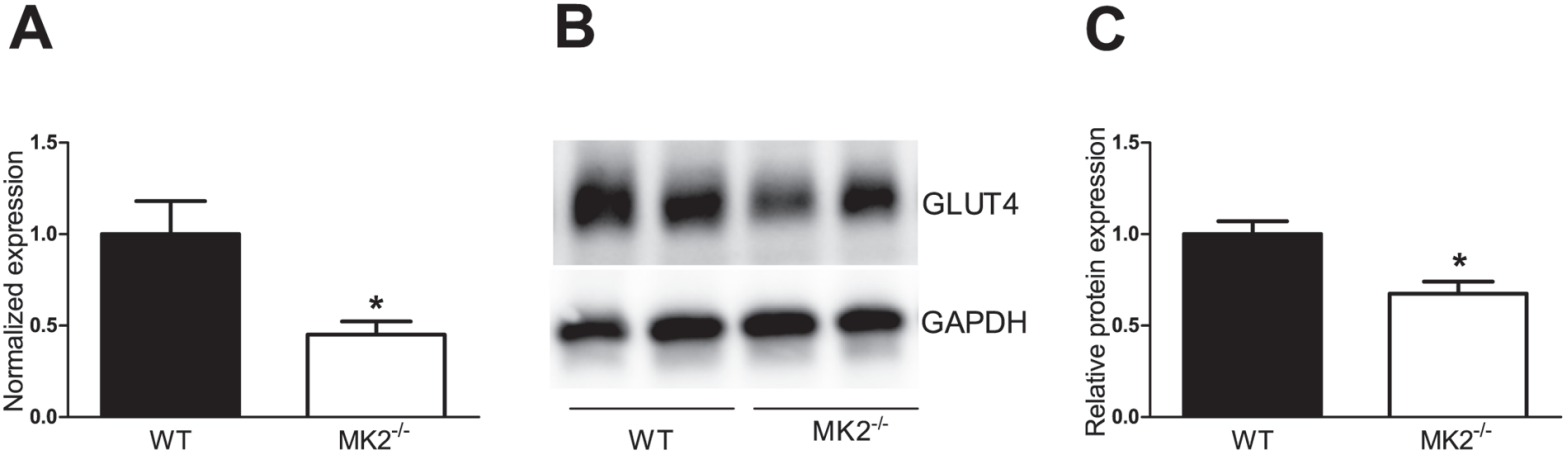
High-fat diet-fed *MK2^−/−^* mice have reduced adipose tissue expression of the insulin-responsive glucose transporter GLUT4. Adipose tissue *Glut4* mRNA expression (n = 8 mice per group) was measured by real-time qPCR (A). Western blot was performed (n = 5 mice per group) to determine GLUT4 protein expression in adipose tissue; GAPDH served as loading control (B); band intensities were quantified using ImageJ software and normalized to the mean intensity of the wild-type (WT) control mice (C). *p<0.05 compared to WT control mice.

To explore the underlying basis for the decreased glucose tolerance and increased insulin resistance in the *MK2^−/−^* mice, expression of the insulin-responsive glucose transporter GLUT4 was determined in the tissues that account for the major part of glucose disposal within the body, namely muscle and adipose tissue. Whereas no difference was found for expression of *Glut4* in muscle (data not shown), *Glut4* mRNA expression was 55% (p<0.05) lower in adipose tissue of *MK2^−/−^* mice (Fig. 3A). Also, GLUT4 protein expression was reduced in the adipose tissue of *MK2^−/−^* mice compared to WT controls (−33%, P<0.05, Fig. 3B and C). Decreased expression of GLUT4 in adipose tissue might therefore represent a contributing factor to the glucose intolerant, insulin resistant phenotype of HFD-fed *MK2^−/−^* mice.

## Discussion

This study demonstrates that HFD-fed *MK2^−/−^* mice are more glucose intolerant and insulin resistant compared to the respective WT controls. Although these results were unexpected considering the important role of MK2 in various animal models of inflammatory disease, *MK2^−/−^* mice were found to have decreased adipose tissue expression of the insulin-responsive glucose transporter GLUT4, which might contribute to the observed glucose intolerant, insulin resistant, phenotype in these mice.

Since the observation that TNFα production is increased in adipose tissue of obese individuals [15], the contribution of adipose tissue inflammation to systemic insulin resistance gained substantial interest. Subsequently, macrophages have been identified as major effector cells of adipose tissue inflammation. Obesity is associated with an increased infiltration of macrophages into adipose tissue [4], [5], [16], [17] and an inverse correlation between the number of macrophages present in the adipose tissue and insulin sensitivity was shown [18]–[20]. Intracellular signal transduction plays a decisive role in inflammatory responses and MAPKs are central in many of these signal transduction pathways. p38 belongs to the MAPK superfamily and has important functions in apoptosis as well as transcriptional regulation, but is also a key mediator of inflammatory signaling [21]. p38 can be activated by inflammatory cytokines but, interestingly, also by free fatty acids and high concentrations of glucose [22], factors that are usually elevated in patients with type 2 diabetes. As p38 has many down-stream targets, its activity affects a wide range of biological processes, which is exemplified by the fact that *p38α^−/−^* mice die in utero [23]. Furthermore, hepatotoxicity has been demonstrated for many p38-inhibitors [24], hampering the clinical use of such compounds. Inhibiting more downstream mediators in the p38 signaling cascade is likely to increase the specificity of the effects and is therefore considered a more attractive therapeutic option [25]. MK2 is a direct target of p38 and is essential for sustained NF-κB activation [26]. Of note, in contrast to *p38α^−/−^* mice, *MK2^−/−^* mice are viable and do not display any obvious health problems [12]. *MK2^−/−^* mice are resistant to LPS-induced endotoxic shock [12] and studies using these mice have indicated that MK2 inhibition might be a good treatment option for a variety of chronic inflammatory diseases [13], [26], [27]. Using a mouse model of collagen-induced RA, it was shown that *MK2^−/−^* mice were protected against disease development [27]. In another study, we previously demonstrated that *MK2^−/−^* mice on the *Ldlr^−/−^* background were protected against atherosclerosis when fed an atherogenic diet [13]. Decreased foam cell formation but also a reduction of macrophage recruitment were identified as mechanisms accountable for the anti-atherogenic phenotype [13]. Since diet-induced obesity as well as insulin resistance coincide with chronic inflammation, and macrophage recruitment to adipose tissue is a critical step in the development of insulin resistance [28], [29], we tested whether MK2-deficiency would ameliorate diet-induced adipose tissue inflammation and would thereby improve insulin sensitivity. Surprisingly, adipose tissue expression of inflammatory cytokines was not markedly different between HFD-fed *MK2^−/−^* and WT mice. Also, the amount of adipose tissue macrophages remained unchanged. However, the subpopulation of Cd11c macrophages, reported to be recruited to adipose tissue in obesity and to have deleterious effects on insulin sensitivity [30], appeared to be increased in the adipose tissue of MK2^−/−^. In adition, the M1/M2 balance seems to be more skewed towards the M1 phenotype in those mice. Expression levels of the polarization markers were normalized to *Cd68* rather than cyclophilin to correct for the number of macrophages present in the adipose tissue. This is justifiable because expression of *Mgl-1* and *Mgl-2* is barely detectable in adipocytes (data not shown). The altered characteristics of adipose tissue macrophages could contribute to the glucose intolerant, insulin resistant phenotype in MK2^−/−^ mice. However, the analysis of macrophage polarization was limited to mRNA expression in the current study. As bone marrow-derived MK2^−/−^ macrophages did not show differences in macrophage polarization (unpublished observations), the results of this study indicate that the specific environment of adipose tissue impacts on macrophage skewing. Additional information could be obtained by studying the characteristics of macrophages isolated from adipose tissue. In addition, adipose tissue contains a wide variety of immune cells that were not quantified in this study. Detailed analysis of all immune cells present in the adipose tissue, e.g. using flow cytometry, could further increase our knowledge regarding the impact of MK2-deficiency on adipose tissue inflammation.

Interestingly, glucose tolerance tests revealed that HFD-fed *MK2^−/−^* mice had impaired glucose tolerance compared to WT controls. In addition, the response during the insulin tolerance test indicated that HFD-fed *MK2^−/−^* mice were significantly more insulin resistant. Fasting hyperglycemia and more than 50% elevated plasma fasting insulin levels further substantiated the conclusion that these mice were insulin resistant. This unexpected phenotype suggests that MK2 not only plays a role in inflammation, but also is a mediator of metabolism. We identified decreased expression of the insulin-responsive glucose transporter GLUT4 in adipose tissue as a mechanism likely contributing to the glucose intolerant, insulin resistant phenotype. With respect to human pathophysiology, GLUT4 expression was found to be decreased in adipose tissue, but importantly not in muscle, of obese individuals and in insulin resistant and type 2 diabetic patients [31], [32]. In addition, the pivotal role of adipose tissue GLUT4 in maintaining insulin sensitivity is emphasized by the fact that adipose tissue-specific *Glut4^−/−^* mice develop insulin resistance [33] whereas mice overexpressing GLUT4 specifically in the adipose tissue have enhanced glucose tolerance and reduced fed insulin levels, indicative of improved insulin sensitivity [34]. Furthermore, overexpression of GLUT4 in adipose tissue of mice that lack GLUT4 expression in muscle corrected the insulin resistant phenotype in those mice [35]. Combined these studies identify adipose tissue GLUT4 expression as a central mediator of whole body glucose tolerance. Accordingly, decreased adipose tissue expression of GLUT4 in *MK2^−/−^* mice conceivably offers an explanation for the glucose intolerant, insulin resistant phenotype observed in our present study. However, as decreased expression of GLUT4 in adipose tissue likely leads to insulin resistance in other organs, insulin signaling and uptake of 2-deoxyglucose in multiple tissues, including muscle and liver, could be assessed in future studies. Furthermore, it remains to be explored how MK2-deficiency leads to decreased adipose tissue GLUT4 expression. In that respect, it is important to point out that only total body MK2-deficient mice were used in the current study. More mechanistic insights might be gained from tissue-specific knock-out mice and/or bone marrow transplantation studies.

The fact that plasma cholesterol levels were increased in *MK2^−/−^* mice further indicates that, in addition to its role in inflammation, MK2 also impacts metabolism. The more dyslipidemic phenotype observed in the HFD-fed *MK2^−/−^* mice in our study is in accordance with our previous data obtained in Western diet-fed *MK2^−/−^ Ldlr^−/−^* mice that had increased plasma levels of apoB-containing lipoproteins [13]. The mechanistic basis underlying the decreased circulating free fatty acids in the HFD-fed *MK2^−/−^* mice remains to be elucidated. As the phenotype of adipose tissue macrophages seems to be different in MK2^−/−^ mice, it is tempting to speculate that they might have an altered capacity to buffer excess circulating lipids. This property of adipose tissue macrophages was recently described [36]. Moreover, it has been demonstrated that p38, the MAPK that activates MK2, has ample effects on glucose and lipid metabolism [22], [37]. Part of these effects might be mediated via MK2, however, the effects of MK2 on metabolic pathways have thus far barely been explored and warrant more detailed investigation.

In conclusion, HFD-fed *MK2^−/−^* mice have increased plasma triglyceride and cholesterol levels and display a glucose intolerant, insulin resistant phenotype. Decreased adipose tissue expression of GLUT4 and an altered macrophage polarization balance in adipose tissue might contribute to this unfavorable metabolic profile. These data indicate that the clinical use of MK2 inhibitors for the treatment of chronic inflammatory diseases has to be evaluated with caution, taking potential metabolic adverse effects into account.

## Supporting Information

Figure S1 High-fat diet-fed MK2^−/−^ mice have increased apoB-containing lipoproteins. Blood was collected at time of sacrifice after 4 hours of fasting and pooled plasma fractions (n = 8 mice per group) were subjected to fast protein liquid chromatography (FPLC) gel filtration using a Superose 6 column as detailed in materials and methods. Subsequently, individual fractions were assayed for cholesterol (A) and triglyceride (B) content. The black line represents the wild-type (WT) mice and the dashed line the MK2^−/−^ mice.
(PDF)

Figure S2 Liver histology of high-fat diet-fed MK2-KO mice and controls shows no obvious difference in steatosis. Formalin-fixed paraffin-embedded sections were stained with hematoxylin and eosin and images were aquired using the Aperio scanning system. Four representative images are shown out of n = 8 mice per group.
(PDF)

Figure S3 Tendency towards decreased glucose tolerance in chow-fed MK2^−/−^ mice. Chow-fed wild-type (WT) and MK2^−/−^ mice were injected intraperitoneally (i.p.) with glucose (1.25 g/kg) and blood glucose levels were measured at the indicated time points (A). Baseline-substracted calculation of the area under the curve (AUC) of the glucose levels during the glucose tolerance test (B). *p<0.05 vs WT control.
(PDF)

Table S1 Expression of inflammation-related genes in chow- and high-fat diet-fed MK2-KO mice and controls.
(PDF)

Table S2 Hepatic expression of inflammation-related genes in High-fat diet-fed MK2-KO mice and controls.
(PDF)
