# Supplementary figures and images for: Mitogen-Activated Protein Kinase-Activated Protein Kinase 2 Deficiency Reduces Insulin Sensitivity in High-Fat Diet-Fed Mice
Source: PLoS One. 2014 Sep 18;9(9):e106300. doi: 10.1371/journal.pone.0106300 (PMC4169416; doi:10.1371/journal.pone.0106300)

Figure S1.

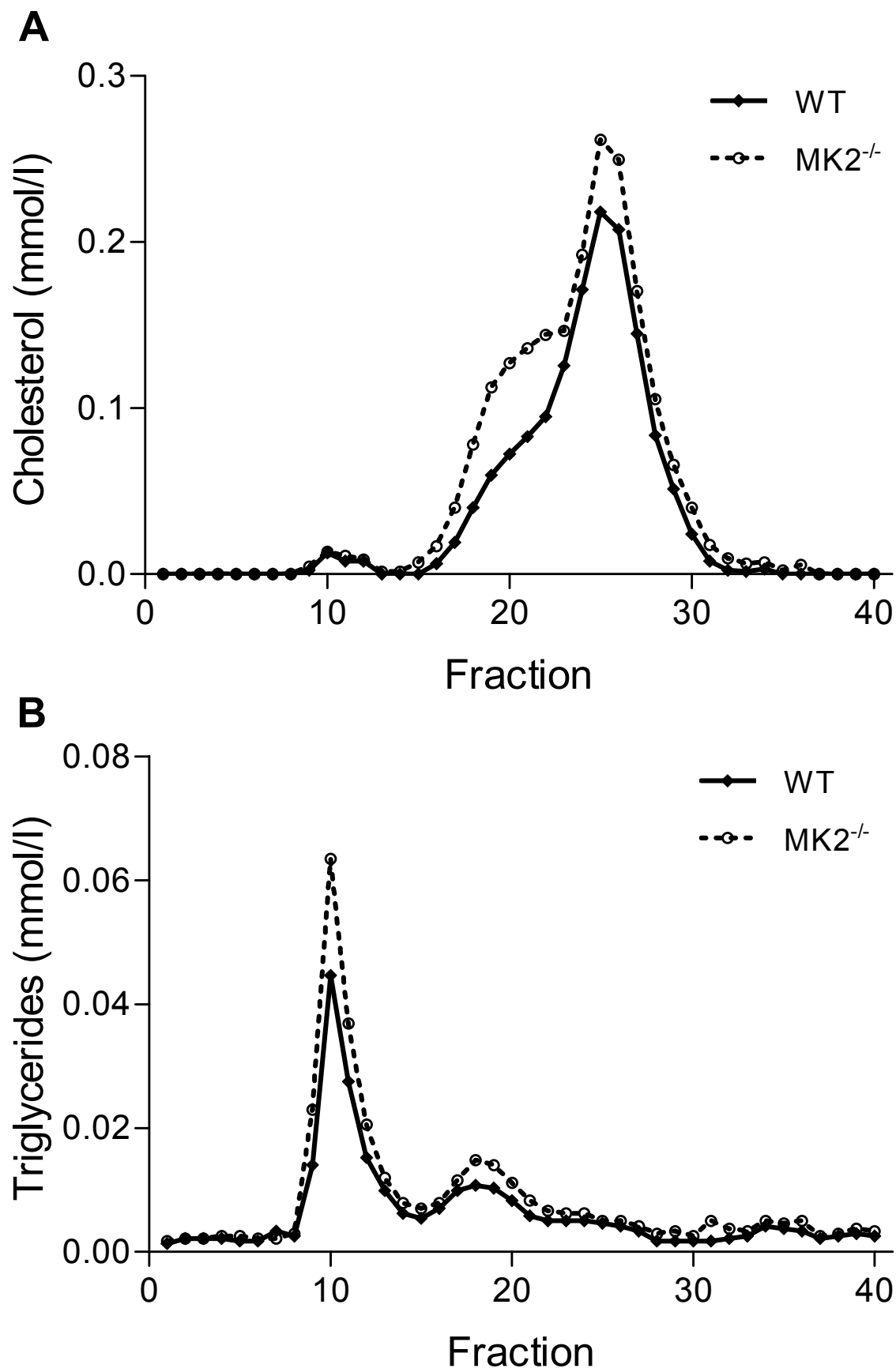

Supplement: Figure S1 — High-fat diet-fed MK2−/− mice have increased apoB-containing lipoproteins. Blood was collected at time of sacrifice after 4 hours of fasting and pooled plasma fractions (n = 8 mice per group) were subjected to fast protein liquid chromatography (FPLC) gel filtration using a Superose 6 column as detailed in materials and methods. Subsequently, individual fractions were assayed for cholesterol (A) and triglyceride (B) content. The black line represents the wild-type (WT) mice and the dashed line the MK2−/− mice. (PDF) [file pone.0106300.s001.pdf]

**Figure S2.**

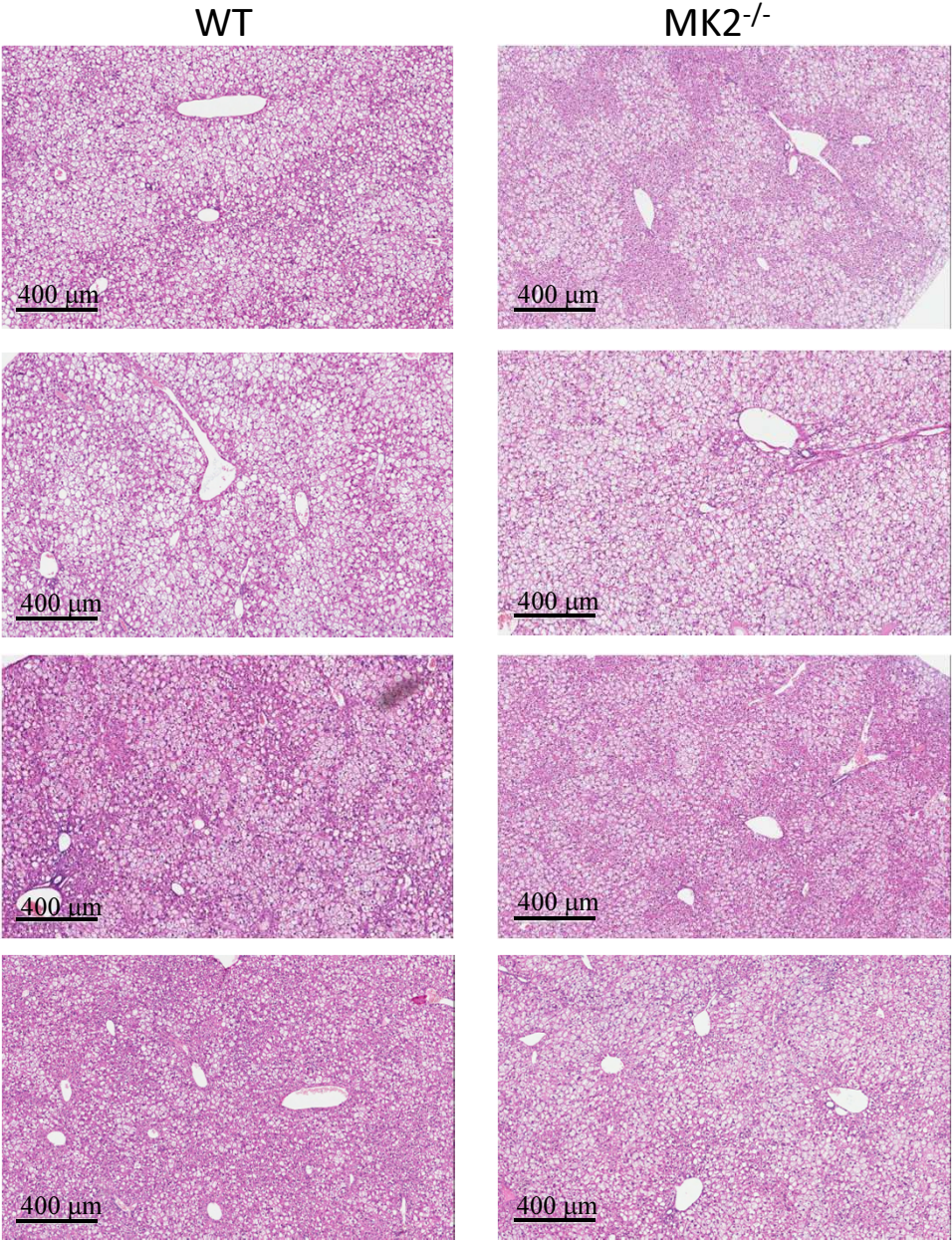

Supplement: Figure S2 — Liver histology of high-fat diet-fed MK2-KO mice and controls shows no obvious difference in steatosis. Formalin-fixed paraffin-embedded sections were stained with hematoxylin and eosin and images were aquired using the Aperio scanning system. Four representative images are shown out of n = 8 mice per group. (PDF) [file pone.0106300.s002.pdf]

Figure S3.

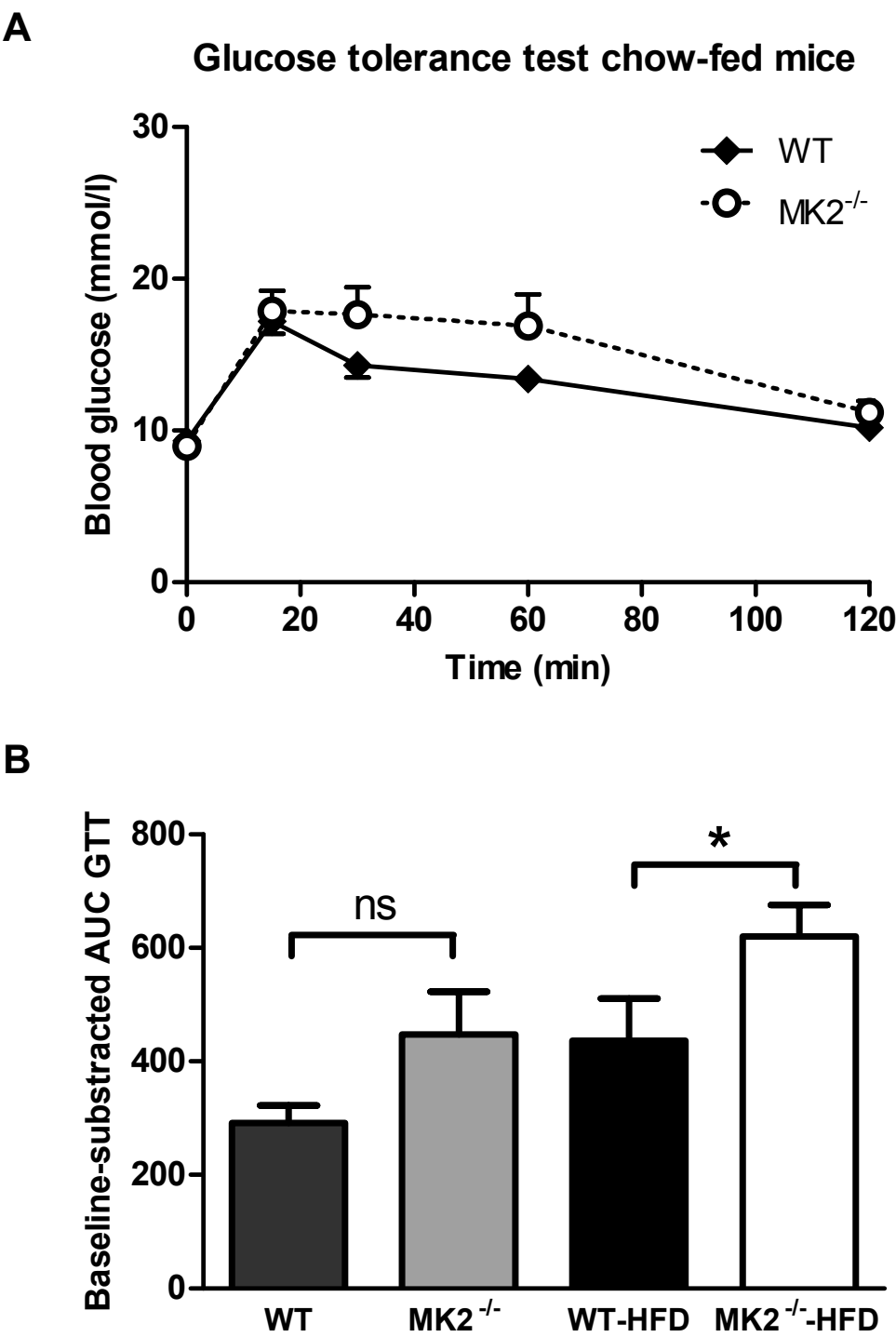

Supplement: Figure S3 — Tendency towards decreased glucose tolerance in chow-fed MK2−/− mice. Chow-fed wild-type (WT) and MK2−/− mice were injected intraperitoneally (i.p.) with glucose (1.25 g/kg) and blood glucose levels were measured at the indicated time points (A). Baseline-substracted calculation of the area under the curve (AUC) of the glucose levels during the glucose tolerance test (B). *p<0.05 vs WT control. (PDF) [file pone.0106300.s003.pdf]
