# Supplementary material for: Mitogen-Activated Protein Kinase-Activated Protein Kinase 2 Deficiency Reduces Insulin Sensitivity in High-Fat Diet-Fed Mice
Source: PLoS One. 2014 Sep 18;9(9):e106300. doi: 10.1371/journal.pone.0106300 (PMC4169416; doi:10.1371/journal.pone.0106300)
Supplement: Table S1 — Expression of inflammation-related genes in chow- and high-fat diet-fed MK2-KO mice and controls. (PDF) [file pone.0106300.s004.pdf]

Table S1. Expression of inflammation-related genes in chow- and high-fat diet-fed MK2-KO mice and controls.

	WT-chow	$MK2^{-/-}$chow	WT-HFD	$MK2^{-/-}$-HFD
Cd68	1.00 ± 0.14	4.02 ± 1.60	6.28 ± 0.91§§§	8.63 ± 0.69§§
F4/80	1.00 ± 0.13	3.38 ± 1.38	5.63 ± 0.95§§§	8.12 ± 0.82§§
Cd11c	1.00 ± 0.32	6.98 ± 3.19*	7.24 ± 1.44§§	14.58 ± 1.87*,§§
Cd3e	1.00 ± 0.18	1.20 ± 0.20	1.21 ± 0.29	2.00 ± 0.31*
Mcp-1	1.00 ± 0.32	2.17 ± 0.48*	2.20 ± 0.35§	2.11 ± 0.12
Tnfα	1.00 ± 0.10	1.70 ± 0.45	2.25 ± 0.22§§	2.97 ± 0.19§§
Il-1β	1.00 ± 0.07	1.60 ± 0.42	1.92 ± 0.37	2.00 ± 0.26
Il-6	1.00 ± 0.11	0.94 ± 0.09	1.18 ± 0.16	1.48 ± 0.10§§
Il-10	1.00 ± 0.18	2.46 ± 0.86	3.27 ± 0.36§§	4.83 ± 0.47§§
Nos2	1.00 ± 0.14	0.71 ± 0.14	0.67 ± 0.07	0.55 ± 0.06
Mgl-1	1.00 ± 0.08	0.54 ± 0.11**	0.37 ± 0.03§§§	0.25 ± 0.01*,§§
Mgl2	1.00 ± 0.06	0.46 ± 0.15**	0.12 ± 0.03§§§	0.05 ± 0.00**,§§§

* p<0.05, ** p<0.01, *** p<0.001 vs. WT controls. § p<0.05. §§ p<0.01, §§§ p<0.01 vs. respective chow controls. Data are given as mean ± SEM of n = 8 mice per group. Expression levels were normalized to cyclophilin and further normalized to the mean expression level of chow-fed WT animals. Expression levels of Mgl-1 and Mgl-2 were normalized to *Cd68* to correct for the number of macrophages present in adipose tissue. WT, wild-type; HFD, high-fat diet.
