# Supplementary material for: Mitogen-Activated Protein Kinase-Activated Protein Kinase 2 Deficiency Reduces Insulin Sensitivity in High-Fat Diet-Fed Mice
Source: PLoS One. 2014 Sep 18;9(9):e106300. doi: 10.1371/journal.pone.0106300 (PMC4169416; doi:10.1371/journal.pone.0106300)
Supplement: Table S2 — Hepatic expression of inflammation-related genes in High-fat diet-fed MK2-KO mice and controls. (PDF) [file pone.0106300.s005.pdf]

Table S2. Hepatic expression of inflammation-related genes in High-fat diet-fed MK2-KO mice and controls.

	WT	$MK2^{-/-}$
Cd68	1.00 ± 0.08	1.08 ± 0.19
Cd3e	1.00 ± 0.07	2.07 ± 0.32*
Mcp-1	1.00 ± 0.10	0.85 ± 0.10
Tnfa	1.00 ± 0.06	0.86 ± 0.14
Il-1b	1.00 ± 0.07	1.16 ± 0.26
Il-6	1.00 ± 0.17	0.61 ± 0.16
Ifng	1.00 ± 0.15	1.71 ± 0.53
Il-10	1.00 ± 0.09	2.65 ± 0.70
Crp	1.00 ± 0.04	1.04 ± 0.05

[*] $p<0.05$ vs. WT. Data are given as mean ± SEM of n = 8 mice per group.
